# The impact of the COVID-19 pandemic on healthcare service access for the victims of sexual assault

**DOI:** 10.4102/safp.v63i1.5367

**Published:** 2021-10-27

**Authors:** Ramprakash Kaswa

**Affiliations:** 1 Department of Family Medicine and Rural Health, Faculty of Health Sciences, Walter Sisulu University, Mthatha, South Africa

**Keywords:** COVID-19, pandemic, violence against women, sexual assault, lockdown

## Abstract

**Background:**

The coronavirus disease 2019 (COVID-19) pandemic disrupted the provision or exacerbated the existing gap of access to essential healthcare services. An unanticipated effect on access to healthcare services emerged with the introduction of COVID-19 lockdown regulations. Violence against women is prevalent with varying degrees of severity in all spheres of society.

**Methods:**

This study aims to evaluate the impact of the COVID-19 pandemic on the access to healthcare services for the victims of sexual assault in the Mthatha region of South Africa. This is a records review of victims of sexual assault survivors who visited and were treated at the Sinawe TCC at Mthatha Regional Hospital. The data on sexual assault cases at Sinawe TCC were compared with a time-matched control group from 2014 to 2020.

**Results:**

There were 5747 sexual assault cases reported at Sinawe TCC between 01 January 2014 and 31 December 2020. There was a major drop in reported cases at Sinawe TCC during the 2020 year, with only about half (451) of the annual average cases being reported.

**Conclusion:**

The COVID-19 pandemic has an impact on access to healthcare services for the victims of sexual assault survivors in the Mthatha region of South Africa.

## Introduction

In response to the rapid spread of the coronavirus disease 2019 (COVID-19), countries around the globe legislated for the cessation of non-essential services.^1^ With the introduction of lockdowns and social distancing regulations, the unanticipated effects on the access to essential healthcare services soon became apparent.^2,3^

Given this context, access to healthcare services is of particular concern during the COVID-19 pandemic, including access for women subjected to violence.^4^

The violence against women has been greater than assumed during the COVID-19 pandemic.^1^ The United Nations (UN) observed a rise in violence against women during the restrictive measures implemented to control the COVID-19 pandemic.^5^ The UN called it a ‘shadow pandemic within the pandemic’ and on March 27, 2020, urged countries around the globe to take additional measures to prevent this shadow pandemic.^6^

According to a recent report of the World Health Organization (WHO), violence against women in various forms, including physical assault, sexual assault, psychological abuse, economic abuse and stalking, has increased since 2019.^7^ Sexual assault is the worst form of violence against women where victims experience lifelong negative consequences.^8^

South Africa has the highest rate of sexual assault amongst African regions and all ages are affected by this crime.^3^ The estimated prevalence of sexual assault in South Africa varies widely within different geographic regions. The prevalence of sexual assault ranges from 1.5% to 18.8% within and across the provinces.^9^ There is a major gap in the real prevalence of sexual assault and reported cases in South Africa because of the lack of service access, the inadequacy of existing service capacity and poor coverage. The reported cases represent the tip of the iceberg as many cases are unreported.^10^ This gap widened during the COVID-19 pandemic lockdown because of the changes in service provision of shelters, support services, community-based agencies and emergency departments.^11^

Across the country, the management of victims of sexual assault occurs in specialised ‘one stop’ treatment centres. These specialised centres have been adopted by many health facilities in South Africa and are known as the Thuthuzela Care Centres (TCCs).^8^ These centres are working in close collaboration with the National Prosecuting Authority (NPA). There are 54 TCCs distributed across the country and they provide comprehensive management of sexual assault victims.^12^ Here we study the access to healthcare services for a victim of sexual assault following the introduction of the COVID-19 lockdown. This study aimed to evaluate the impact of the COVID-19 pandemic on the access to healthcare services for the victims of sexual assault survivors in the Mthatha region of South Africa. The objective of this study was to access the effect of the COVID-19 pandemic on sexual assault cases admitted at Sinawe TCC in Mthatha Regional hospital compared with time-matched control group from 2014.

## Methods

This is a records review of victims of sexual assault who visited and were treated at the Sinawe TCC, which is attached with Mthatha Regional Hospital. The Sinawe TCC is providing its services to a population of about 500 000 of the Oliver Tambo District Municipality, which includes Mthatha, Mquanduli, Ngqeleni, Libode, Tsolo and Engcobo. The centre offers 24/7 clinical care to victims of sexual assault. It is a ‘One Stop Centre’ providing multidisciplinary management of victims of sexual assault. Survivors who report to the centre following sexual assault undergo an emergency medical assessment, collection of medico-legal evidence and counselling. There is a service provision of post-exposure prophylaxis (PEP), which includes prevention of pregnancy, sexually transmitted infections, Hepatitis-B and HIV, within 72 h following the sexual assault. This is followed by further visits for review and monitoring of PEP, pregnancy screening, contraception choices and counselling at 1 week, 6 weeks and 3 months.^10^

The data were manually extracted from the admission records of the Sinawe TCC of the Mthatha Regional Hospital. An Excel spreadsheet was used to extract the data from 01 January 2014 to 31 December 2020. All data collected from the records were cross-checked with the hospital database for accuracy and quality assurance. The data analysis was performed with the Statistical Package for Social Sciences (SPSS) V.18 and results were demonstrated in tables and graphs. The data on sexual assault cases at Sinawe TCC were compared with a time-matched control group from 01 January 2014 to 31 December 2020.

### Ethical consideration

Ethical clearance was obtained from the Human Research and Ethics Committee of the Walter Sisulu University (Reference number: 098/2020). Permission to conduct the study was also obtained from the Eastern Cape Department of Health and Hospital Management (Reference number: EC_202010_027).

## Results

There were 5747 sexual assault cases reported at Sinawe TCC between 01 January 2014 and 31 December 2020. Annually, an average of 882.6 sexual assault case was reported at Sinawe TCC between 01 January 2014 and 31 December 2019.

**FIGURE 1:**
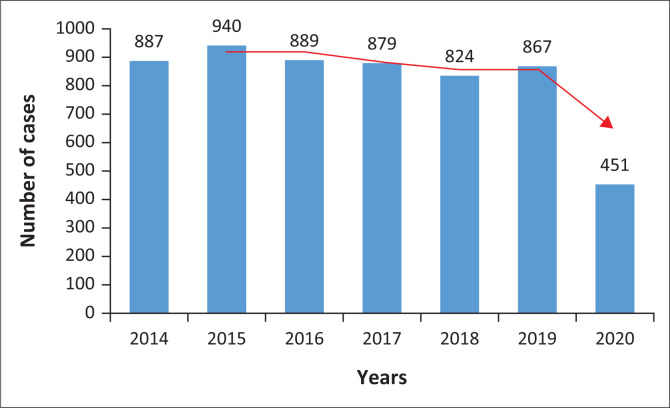
Annual distribution of sexual assault cases reported between 01 January 2014 and 31 December 2020 at Sinawe Thuthuzela Care Centre, Mthatha.

**FIGURE 2:**
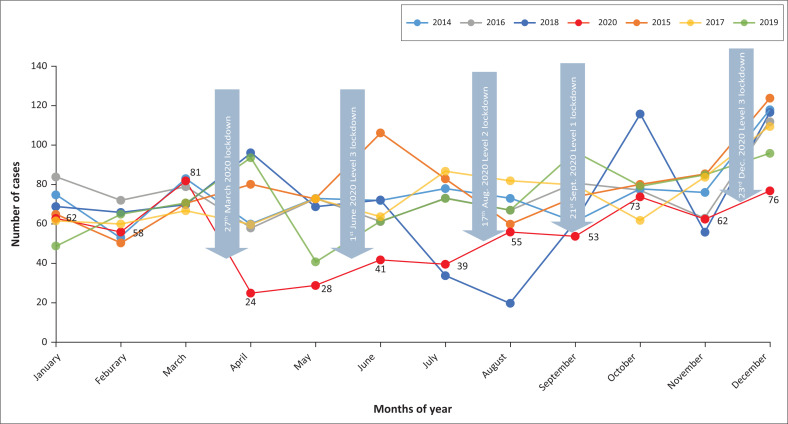
Monthly distribution of sexual assault cases at Sinawe Thuthuzela Care Centre, Mthatha.

There was a major drop in reported cases at Sinawe TCC during the 2020 year where only about half (451) of the annual average cases were reported. Figure 1 demonstrates the number of sexual assault cases reported between 01 January 2014 and 31 December 2020. A major drop in reported cases was observed during April 2020 immediately after the first lockdown was announced in South Africa. Figure 2 demonstrates the monthly distribution of the number of cases reported during different stages of the COVID-19 lockdown.

## Discussion

Sexual assault occurs in all regions across the globe and the incidences are commonly under-reported under normal circumstances.^6^ The reporting of sexual assault incidents has further deteriorated under COVID-19 lockdown.^1^ The disruption of protective social networks, stress and decreased access to services can increase the vulnerability of women to sexual violence.^11,13^ The current pandemic has placed immense pressure on healthcare workers and health systems taking care of COVID-19-related cases. It has become harder for healthcare workers to appropriately screen for sexual and gender-based violence.^2,6^ Our study’s findings demonstrated sudden drops in reporting of sexual assaults after the announcement of the COVID-19 lockdown in South Africa. However, the reporting improved once lockdown levels moved from level-5 to level-1 and it reached almost normal reporting during October 2020 when the lockdown was at level-1.

Although there was a dramatic decrease in reported cases of sexual assault during the year 2020, this data must be interpreted within the context of the COVID-19 pandemic. A decrease in reporting of sexual assault cases may not imply a decrease in the number of incidents but could be an increase in difficulty in accessing the services during the COVID-19 pandemic.^14^ It may also be because of the scaling back of support systems such as civil society, crisis centres, helplines, shelters and other frontline organisations of community response during the pandemic lockdown.^7^

Recent reports indicate that sexual assault incidences increased during the COVID-19 pandemic.^6^ The measures put in place to address the pandemic, such as staying home and physical distancing, have likely increased the risk of women and girls experiencing sexual violence.^15^ At the same time, health services and police, who are the first responders for these incidences, are overwhelmed, have reprioritised, or are unavailable to respond during the pandemic. Community support groups and non-profit organisations that support the vulnerable women to society are affected in term of human resource capacity and financial sustainability by lockdown regulations.^14,16^

Emerging data from parts of Asia, America and Europe show a significant increase in reporting of sexual assault during the current pandemic.^5^ As a result of its sudden surge during the COVID-19 pandemic, on March 27, 2020, abuse against women was pronounced by the UN as a ‘shadow pandemic within the pandemic’.^6^ About three-quarters (76%) of frontline services from the United Kingdom have been reduced as healthcare workers are overburdened and prioritising the COVID-19 cases.^5^ Access to critical care for the survivors of sexual assault was disrupted during the pandemic. The data from Tunisia reported a fivefold increase in calls to a helpline within a few days of the implementation of the COVID-19 pandemic lockdown measures.^7^ Kenya’s National Research Centre reported increased sexual violence against children during the COVID-19 pandemic. The report also highlighted the obstacles and risks of reporting sexual assault cases during the pandemic.^16^ A recent report also highlighted the surge of violence against women in South Africa during the COVID-19 lockdown and stated that one woman was killed every 3 h. Violence against women during the COVID-19 pandemic is described as a ‘twin pandemic’.^3^

The lockdown regulations of the COVID-19 pandemic have raised multiple concerns, especially for their impact on essential services access to victims of sexual assault.^17^ Many researchers and international organisations have argued that lockdown regulations would increase violence against women.^4,15,18^ A national response needs to include an integrated operation plan by the health, security, social welfare and justice sectors for better access to these essential services and limitation of the violence against women, especially sexual offenses, during the current COVID-19 pandemic.^9^

## Limitation of study

This study reported about the data from a single TCC centre in a rural setting and the findings of the study cannot be generalised. There is a possibility that some sexual assault victims received healthcare services elsewhere. In addition, the data were collected from the clinical record and they could not be verified independently that the decrease in reporting was because of poor access or decreased incidence of sexual assault during COVID-19 lockdown.

## Conclusion

The COVID-19 pandemic has an impact on access to healthcare services for the victims of sexual assault in the Mthatha region of South Africa. There is a need for an integrated service as part of the COVID-19 response plans to respond to the violence against women. There is also a need for community-wide engagement so that essential healthcare services are not suspended during periods of COVID-19 lockdown.
